# Respiratory Syncytial Virus-Associated Severe Acute Respiratory Infections in Hospitalized Patients at a University Hospital Center in Rabat, Morocco: An Epidemiological Overview

**DOI:** 10.3390/v18050530

**Published:** 2026-04-30

**Authors:** Ghizlane El-Amin, Naima El Hafidi, Soumia Benchekroun, Mahraoui Chafiq, Amal Zouaki, Nora Touyar, Najat Bouihat, Salma Ech-Cherif El Kettani, Saad Harrak, Larbi Ed-Dafali, Aziza Bentalha, Mustapha Alilou, Hamza El Hamzaoui, Amina Barkat, Ilham Elouardighi, Tarek Dendane, Khalid Abidi, Jihane Bel Ayachi, Naoufal Madani, Redouane Abouqal, Hicham Harmouche, Mouna Maamar, Rachid El Jaoudi, Mourad Feindiri, Myriam Seffar, Mohamed Bouskraoui, Hakima Kabbaj

**Affiliations:** 1 Central Laboratory of Virology, Faculty of Medicine and Pharmacy, Ibn Sina University Hospital Center, Mohammed V University, Rabat 10000, Moroccokabbaj_hakima@yahoo.fr (H.K.); 2 Department of Pediatric Infectious Diseases and Pneumo-Allergology, Children’s Hospital, Faculty of Medicine and Pharmacy, Ibn Sina University Hospital Center, Mohammed V University, Rabat 10000, Morocco; 3 Pediatric Anesthesia and Intensive Care Unit, Children’s Hospital, Faculty of Medicine and Pharmacy, Ibn Sina University Hospital Center, Mohammed V University, Rabat 10000, Morocco; 4 Emergency Department, Faculty of Medicine and Pharmacy, Ibn Sina University Hospital Center, Mohammed V University, Rabat 10000, Morocco; 5 Neonatology Department, Children’s Hospital, Rabat, Ibn Sina University Hospital, Mohammed V University in Rabat, Rabat 10000, Morocco; 6 Infectious Diseases Research Laboratory, Faculty of Medicine and Pharmacy, Cadi Ayyad University, Marrakech 40000, Morocco; 7 Ibn Sina University Hospital, Mohammed V University, Rabat 10000, Morocco; 8 Medical Intensive Care Unit, Faculty of Medicine and Pharmacy, Ibn Sina University Hospital Center, Mohammed V University, Rabat 10000, Morocco; 9 Hospital Emergency Department, Faculty of Medicine and Pharmacy, Ibn Sina University Hospital Center, Mohammed V University, Rabat 10000, Morocco; 10 Internal Medicine Department, Clinical Immunology, Ibn Sina Hospital, Rabat, Morocco, Faculty of Medicine and Pharmacy, Mohammed V University, Rabat 10000, Morocco; 11 Pharmacology-Toxicology Laboratory, Faculty of Medicine and Pharmacy, Ibn Sina University Hospital Center, Mohammed V University, Rabat 10000, Morocco

**Keywords:** respiratory syncytial virus, SARI, multiplex PCR, seasonality, co-infection, Morocco, post-COVID-19

## Abstract

Respiratory syncytial virus (RSV) imposes a substantial burden of severe acute respiratory infection (SARI), especially in young children and the elderly. Methods: We describe RSV epidemiology among hospitalized SARI patients at the Ibn Sina University Hospital Center (Rabat, Morocco) from 1 January 2021, to 31 December 2025, using multiplex PCR (BioFire^®^ RP2.1plus or Xpert^®^ SARS-CoV-2/Flu/RSV). Results: Among 4604SARI samples, RSV prevalence was 16.1% (739/4604), predominantly pediatric (88.6%, *p* < 0.001), with peak burden in infants <6 months (70.4% of cases, *p* < 0.001). Pediatric prevalence was 28.3% (655/2316) vs. 3.8% (84/2204) in adults (*p* < 0.001), with predominance in the elderly ≥60 years (51/1041, 4.9%). Co-infections occurred in 46.7% (310/665) of FilmArray-tested positives (total = 665), led by rhinovirus/enterovirus (198/310, 63.9%), and were significantly higher in children (48.5%, *p* < 0.001). RSV peaked in winter (51.6%), except for summer dominance in 2021 (52.5%), reflecting COVID-19 non-pharmaceutical intervention effects. Conclusions: These data establish Morocco’s first comprehensive RSV surveillance baseline, highlighting post-pandemic epidemiological shifts. As maternal vaccines and monoclonal antibodies emerge, these data inform optimal implementation in low- and middle-income countries (LMICs).

## 1. Introduction

Respiratory Syncytial Virus (RSV) is an enveloped, single-stranded, negative-sense RNA virus belonging to the genus Orthopneumovirus within the family Pneumoviridae and is responsible for a major respiratory infection that primarily affects epithelial cells of the upper and lower respiratory tract.

Since its identification in 1955, RSV has been recognized as a leading cause of acute lower respiratory tract infections, particularly in infants and young children [1,2,3,4], with more than 33 million RSV-associated lower respiratory tract infection episodes and 3.3 million hospitalizations annually in children under 5 years of age, nearly 95% of which occur in low- and middle-income countries [2,5,6,7].

Beyond pediatrics, RSV is increasingly recognized as a clinically relevant pathogen in adults, particularly in elderly individuals and in those with chronic cardiopulmonary disease or other comorbidities [8,9,10,11,12]. In elderly populations, it is among the three most common respiratory pathogens, alongside influenza and rhinovirus. Globally, most severe RSV infections requiring hospitalization are reported in infants younger than 6 months, as well as in older adults and immunocompromised individuals, underscoring the pivotal role of immunity acquired during primary infection in protecting against subsequent severe disease [2,4,5,7,11,12,13].

Several studies have demonstrated that RSV reinfection is common throughout life, reflecting incomplete and short-lived immunity. Additional factors, including age at primary exposure, immune maturation, and age-related physiological changes, also play a key role in determining the frequency of reinfection. The clinical impact of RSV infection varies markedly across populations, with frequent reinfections that are typically mild or asymptomatic in immunocompetent young adults but which result in clinically significant disease in older individuals, a vulnerability likely driven by immunosenescence and the accumulation of comorbidities [11,14,15,16].

RSV is classically divided into two major antigenic subgroups, RSV-A and RSV-B, each comprising multiple genotypes [17,18,19,20]. This virus exhibits substantial genetic diversity and dynamic phylodynamic evolution, largely driven by variability in the attachment glycoprotein G, the most heterogeneous region of the viral genome and a key determinant of antigenic diversification [17,18,19,20]. In this context, two major duplication genotypes have profoundly shaped recent RSV molecular epidemiology: the RSV-B BA lineage, first identified around 1999 in Buenos Aires and characterized by a 60-nucleotide duplication in the second hypervariable region of the G gene, and the RSV-A ON1 genotype, first detected in Ontario in 2010 and defined by a 72-nucleotide duplication in the same region [17,19,21,22,23,24]. These ON1 and BA lineages spread rapidly worldwide and progressively replaced previously circulating strains, suggesting a selective fitness advantage and a marked capacity to adapt to host immune pressures [17,19,21,22,23,24]. Phylodynamic analyses indicate that the global genetic landscape of RSV has become increasingly dominated by ON1-derived RSV-A and BA-derived RSV-B viruses, with marked temporal and geographic variation, frequent co-circulation of RSV-A and RSV-B, and alternating subgroup predominance across successive epidemic seasons [18,19,20,22,23,24,25]. More recent genomic and epidemiological data further suggest that the post-2022 resurgence of RSV has been accompanied by the continued predominance and diversification of ON1- and BA-derived lineages, underscoring the importance of sustained molecular surveillance to monitor viral evolution and to inform the implementation of new vaccine and monoclonal antibody strategies [24,25,26].

In the post-pandemic context, RSV surveillance has taken on renewed importance. Changes in herd immunity, mitigation measures, healthcare-seeking behaviours and viral interference have disrupted traditional seasonal epidemiological patterns. In this setting, robust surveillance systems are essential to (i) support public health planning and evidence-based resource allocation; (ii) anticipate the timing and intensity of seasonal peaks; and (iii) inform vaccination strategies, as RSV vaccines and long-acting monoclonal antibodies are progressively deployed in Europe and other regions, including the evaluation of their potential extension to high-risk adult populations [3,8,9,26,27,28].

The declaration by the World Health Organization on 5 May 2023 of the end of the public health emergency related to the COVID-19 pandemic marks a pivotal transition, ushering in a new phase in global RSV epidemiology characterized by evolving and less predictable transmission dynamics.

Respiratory syncytial virus was already well established as a major respiratory pathogen in Morocco before the COVID-19 pandemic, particularly among children, with reported prevalence estimates ranging from approximately 12% to 36.5%, depending on the study population and diagnostic setting [29,30,31,32,33]. In a prospective study conducted at the Hôpital d’Enfants de Rabat between November 2010 and December 2011, 700 children aged 2–59 months with WHO-defined severe pneumonia were enrolled, and RSV was identified in 18% of cases. Similarly, a hospital-based study from Mohammed VI University Hospital in Marrakech, conducted between January 2018 and December 2019, detected RSV in 124 of 534 hospitalized children aged under 14 years (23.2%). Within the same institution, a pre-pandemic study by Marcil et al., carried out between October 2015 and August 2016, reported RSV A/B detection in 12% of severe acute viral respiratory infections. A recent national systematic review of Moroccan data further confirmed that RSV is one of the most frequently detected viral pathogens in severe acute respiratory infections across different regions of the country. In parallel, global pre-pandemic evidence, including the PERCH (Pneumonia Etiology Research for Child Health) study and more recent reviews, consistently identified RSV as the leading viral cause of radiologically confirmed pneumonia among hospitalized young children. In our setting, a retrospective study conducted at the Central Virology Laboratory of Ibn Sina University Hospital in Rabat, including all patients hospitalized for acute respiratory tract infection and tested by a multiplex respiratory PCR panel between 1 January and 31 December 2021, showed that, among RSV-positive cases, children accounted for 80% (36/45), whereas adults represented 20% (9/45).

Although maternal vaccines and long-acting monoclonal antibodies against RSV are now becoming available, their effective deployment remains highly context-dependent. In low- and middle-income settings, including Morocco, critical gaps persist in baseline epidemiological data, particularly regarding age-specific disease burden, seasonal dynamics, and the impact of recent disruptions associated with the COVID-19 pandemic. Such data are essential to define optimal target populations, determine the appropriate timing of intervention, and ensure efficient allocation of limited healthcare resources [1,3,8,9,26,27,28]. In addition, the pandemic and post-pandemic periods have been characterized by atypical RSV circulation patterns, further limiting the applicability of pre-pandemic data for current public health decision-making.

In this context, the present study aims to characterize the epidemiology of RSV among pediatric and adult inpatients at the Ibn Sina University Hospital Centre in Rabat, Morocco, across both the pandemic and post-pandemic periods, in order to generate locally relevant evidence to inform future prevention strategies.

## 2. Materials and Methods

### 2.1. Study Design, Setting and Study Period

This retrospective observational study was conducted at the Central Virology Laboratory (CVL) of the Ibn Sina University Hospital Center (UHC), a tertiary care referral center in Rabat, Morocco. The hospital primarily serves the Rabat-Salé-Kénitra region, which had a legal population of 5,132,639 inhabitants on 1 September 2024, representing 13.9% of the Moroccan population, according to the 2024 General Population and Housing Census issued by the High Commission for Planning [34]. The study period extended from 1 January 2021 to 31 December 2025, covering both the pandemic and post-pandemic phases.

### 2.2. Study Population and Case Definition

We included all hospitalized patients, both pediatric and adult, for whom a respiratory multiplex polymerase chain reaction (PCR) test was performed at the CVL for a clinical presentation consistent with severe acute respiratory infection (SARI).

### 2.3. Specimen Collection and Transport

Samples were collected using nasopharyngeal swabs and transported to the CVL in Universal Transport Medium UTM^®^-RT, supplied as part of a collection kit, to preserve specimen integrity before laboratory testing. The UTM^®^-RT system was manufactured by Copan Italia S.p.A., Brescia, Italy. Following receipt, the samples were processed in accordance with the laboratory’s established standard operating procedures.

### 2.4. PCR Tests

Samples were tested using either the BIOFIRE^®^ FilmArray^®^ Respiratory Panel 2.1 plus (RP2.1plus; BioFire Diagnostics, LLC, Salt Lake City, UT, USA) or the Xpert^®^ Xpress SARS-CoV-2/Flu/RSV plus assay, performed on GeneXpert^®^ systems (Cepheid, Sunnyvale, CA, USA), depending on the clinician’s request and seasonal viral circulation. Both assays were performed within the laboratory quality framework, including internal controls embedded in each cartridge or pouch, and runs were accepted only when the controls met the manufacturers’ criteria.

#### 2.4.1. BIOFIRE^®^ FilmArray^®^ Respiratory *Panel 2.1 Plus*

The BIOFIRE^®^ FilmArray^®^ Respiratory Panel 2.1 plus, manufactured by BioFire Diagnostics, LLC, Salt Lake City, UT, USA, enables simultaneous detection of 19 viruses, including respiratory syncytial virus (RSV), adenovirus (ADV), coronavirus 229E (CoV 229E), coronavirus HKU1 (CoV HKU1), coronavirus NL63 (CoV NL63), coronavirus OC43 (CoV OC43), Middle East respiratory syndrome coronavirus (MERS-CoV), severe acute respiratory syndrome coronavirus 2 (SARS-CoV-2), human metapneumovirus (hMPV), influenza A virus (IAV), influenza A/H1, influenza A/H1-2009, influenza A/H3, influenza B virus (IBV), parainfluenza viruses types 1 to 4 (PIV1–4), and human rhinovirus/enterovirus (HRV/HEV), as well as four intracellular bacteria: *Bordetella pertussis*, *Bordetella parapertussis*, *Mycoplasma pneumoniae*, and *Chlamydia pneumoniae*. The reaction is based on nested PCR with melting curve analysis. It is a single-use, closed, disposable system containing all reagents required for nucleic acid extraction, amplification, and detection, with results available in approximately 45 min.

#### 2.4.2. Xpert^^®^^ Xpress SARS-CoV-2/Flu/RSV

The Xpert^®^ Xpress SARS-CoV-2/Flu/RSV plus assay, manufactured by Cepheid, Sunnyvale, CA, USA, and performed on GeneXpert^®^ systems, is a multiplex real-time reverse transcription PCR (RT-PCR) assay for the qualitative in vitro detection and differentiation of SARS-CoV-2, influenza A virus, influenza B virus, and RSV. For RSV, the assay targets genomic regions allowing detection of both RSV A and RSV B, with primers and probes designed to amplify specific sequences in the non-structural protein, matrix, and nucleocapsid genes [35].

### 2.5. Data Processing

Data were collected and anonymized using the laboratory information system and exported to Microsoft Excel, version [please insert exact version, e.g., Microsoft Excel 2019 or Microsoft Excel for Microsoft 365] (Microsoft Corporation, Redmond, WA, USA). The dataset was then aligned with patients’ laboratory results. The retained variables included date of birth, PCR result, detected virus or bacterium, hospital, and admission department. Microsoft’s headquarters are in Redmond, Washington, USA.

### 2.6. Statistical Analysis

The statistical analysis was performed using Jamovi^^®^^ version 2.3.28.0 software. Categorical variables were compared using the Chi-square test or Fisher’s exact test, as appropriate. Continuous variables were evaluated using the Mann–Whitney U test or Kruskal–Wallis test based on distribution. A probability (*p*) value of less than 0.05 was considered statistically significant. Qualitative variables: Data were presented as counts and percentages and were compared using either the Chi-square test or Student’s t-test.

For analytical purposes, the demographic, temporal, and clinical variables were classified into predefined categories as follows:

➢ Age groups: Patients were stratified into children vs. adults into the following categories: 0–31 days–1–6 months, 6 months–2 years, >2–5 years, >5–16 years, 17–59 years and ≥60 years.
➢ Seasons were defined according to the astronomical calendar in the northern hemisphere. They are divided as follows: winter (21 December–20 March), spring (21 March–20 June), summer (21 June–20 September), and autumn (21 September–20 December).
➢ The clinical services are structured around several critical care centers were divided into two categories—adult and pediatric—and then distributed as follows. Pediatric services were divided as follows: pediatric intensive care unit, neonatology units, and the infectious diseases and allergy department. The other clinical services were grouped under a single entity, “non-acute medical service pediatric”, comprising the various pediatric units and the pediatric hematology-oncology center. Adult services were organized as follows: adult intensive care included intensive care unit and the adult emergency department. Furthermore, services other than these that were not part of these clinical services were grouped under the category “non-acute medical service adult”.

## 3. Results

### 3.1. Demographic Details of the Enrolled Patients

Between January 2021 and December 2025, a total of 4604 respiratory samples were analyzed. Overall, 55.2% of samples were collected from males (n = 2540) and 44.8% from females (n = 2064), representing a male-to-female ratio of 1.23. Of these, 2316 (50.3%) were from children and 2288 (49.7%) from adults.

Among the 4408 patients with available age data, the mean age was 28.3 ± 30.6 years, and the median age was 10.7 years (range, 0–100.2 years), indicating a broad age distribution driven by the coexistence of a large pediatric population and a substantial number of older adults. The distribution of cases according to age group, hospital service, and season is shown in Table 1.

**Figure 1:**
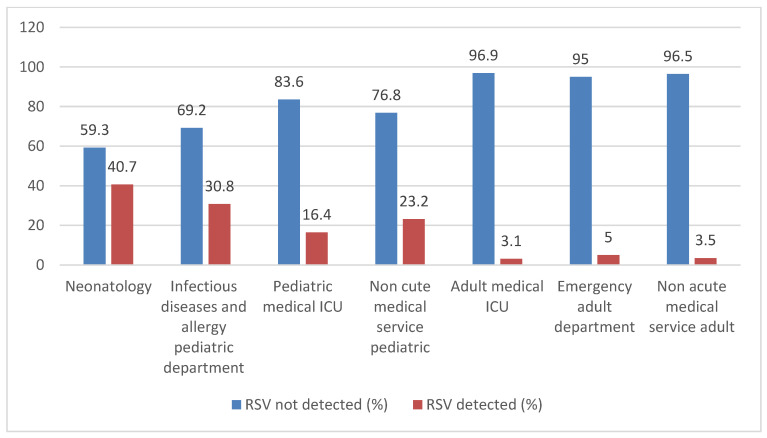
Distribution of RSV-positive and RSV-negative cases across clinical service (Ibn Sina University Hospital Center, Rabat, Morocco, 2021–2025) illustrates the distribution of RSV-positive and RSV-negative cases.

Inferential statistical analyses were performed to compare patient characteristics across study years. No significant difference was observed in sex distribution across years (*p* = 0.363; Cramér’s V = 0.031). In contrast, age category and age group distributions differed significantly (both *p* < 0.001), although with small to moderate effect sizes (Cramér’s V = 0.135 and 0.181, respectively). Seasonal distribution showed a moderate and highly significant variation across years (*p* < 0.001; Cramér’s V = 0.250), representing the strongest association. Similarly, clinical service distribution varied significantly (*p* < 0.001; Cramér’s V = 0.229). Continuous age also differed significantly between years (Kruskal–Wallis *p* < 0.001), indicating changes in the age profile of hospitalized patients over time (Figure 1).

### 3.2. RSV Detection in the Enrolled Patients (Table 2)

Among the 4604 samples analyzed, 739 (16.1%) were RSV-positive. Detection was performed using the FilmArray^^®^^ Respiratory Panel 2.1 plus (BioFire^^®^^) in 665/739 cases (90.0%) and the Xpert^^®^^ Xpress SARS-CoV-2/Flu/RSV assay in 74/739 cases (10.0%). RSV positivity varied significantly across years, from 23.7% (177/748) in 2021 to 16.4% (227/1383) in 2022, 10.1% (107/1057) in 2023, 17.4% (136/781) in 2024, and 14.5% (92/635) in 2025 (*p* < 0.001; Cramér’s V = 0.184 for age-group distribution across years). RSV infections occurred predominantly in children, who accounted for 655/739 cases (88.6%), compared with 84/739 cases in adults (11.4%). The positivity rates were 28.3% in children (655/2316) and 3.7% in adults (84/2288), with 51/1041 adults aged ≥60 years testing positive (4.9%). Among RSV-positive children, median age ranged from 1.0 month in 2024 to 5.0 months in 2021, with an overall median age of 3.0 months (IQR, 1.0–9.0). Among RSV-positive adults, median age ranged from 64.3 to 72.1 years, with an overall median age of 68.7 years (IQR, 54.8–75.7). The distribution of RSV-positive cases by age category did not differ significantly across years (*p* = 0.083; Cramér’s V = 0.106), whereas age-group distribution varied significantly across years (*p* < 0.001; Cramér’s V = 0.184). The highest proportions were consistently observed among infants aged 1–6 months, who represented 33.9% of RSV-positive cases in 2021, 45.4% in 2022, 46.7% in 2023, 47.8% in 2024, and 22.8% in 2025, followed by neonates, who accounted for 13.6%, 22.0%, 13.1%, 25.7%, and 34.8% of yearly RSV-positive cases, respectively. Children aged 6 months to 2 years showed a decline followed by a rebound in 2025, while older pediatric groups contributed fewer cases, with no cases recorded in the 5–16-year group in 2023–2024. Among adults aged 16–59 years, the highest proportion was observed in 2024 (13/136, 9.6%).

**Table 2:** Baseline characteristics of SARI patients with confirmed RSV infection across study periods (Ibn Sina University Hospital Center, Rabat, Morocco, 2021–2025). *Values are n (%)*.

Characteristics	2021 (RSV, n = 177)	2022 (RSV, n = 227)	2023 (RSV, n = 107)	2024 (RSV, n = 136)	2025 (RSV, n = 92)	Total (RSV, n = 739)	*p*-Value	Cramér’s V (95% CI)
RSV detection among all SARI, n (%)	177/748 (23.7%)	227/1383 (16.4%)	107/1057 (10.1%)	136/781 (17.4%)	92/635 (14.5%)	739/4604 (16.1%)	—	—
Gender, n (%)							0.735	0.052 (0.030–0.147)
Female	78 (44.1%)	85 (37.4%)	42 (39.3%)	54 (39.7%)	35 (38.0%)	294 (39.8%)		
Male	99 (55.9%)	142 (62.6%)	65 (60.7%)	82 (60.3%)	57 (62.0%)	445 (60.2%)		
Age category, n (%)							0.083	0.106 (0.057–0.179)
Children	160 (90.4%)	206 (90.7%)	89 (83.2%)	115 (84.6%)	85 (92.4%)	655 (88.6%)		
RSV-positive, n	160	206	89	115	85	655		
Missing age, n	0	1	1	1	1	4		
Age analyzed, n	160	205	88	114	84	651		
Age, median (IQR)	5.0 months (2.0–17.0)	3.0 months (1.0–7.0)	3.0 months (1.0–7.3)	1.0 month (0.0–3.0)	4.5 months (0.0–10.3)	3.0 months (1.0–9.0)		
Min–Max	0–94 months	0–163 months	0–57 months	0–58 months	0–123 months	0–163 months		
Adults	17 (9.6%)	21 (9.3%)	18 (16.8%)	21 (15.4%)	7 (7.6%)	84 (11.4%)		
RSV-positive, n	17	21	18	21	7	84		
Missing age, n	0	0	2	1	2	5		
Age analyzed, n	17	21	16	20	5	79		
Age, median (IQR)	68.7 years (61.0–75.5)	65.8 years (55.0–82.9)	69.6 years (65.0–73.4)	64.3 years (49.0–71.7)	72.1 years (70.9–73.2)	68.7 years (54.8–75.7)		
Min–Max	52.3–91.9 years	25.3–92.9 years	33.0–83.6 years	28.5–93.5 years	28.2–76.1 years	25.3–93.5 years		
Age group, n (%)							<0.001	0.190 (0.180–0.245)
0–31 days	24 (13.6%)	50 (22.0%)	14 (13.1%)	35 (25.7%)	32 (34.8%)	155 (21.0%)		
1–6 months	60 (33.9%)	103 (45.4%)	50 (46.7%)	65 (47.8%)	21 (22.8%)	299 (40.5%)		
6 months–2 years	49 (27.7%)	39 (17.2%)	16 (15.0%)	10 (7.4%)	26 (28.3%)	140 (18.9%)		
>2–5 years	21 (11.95%)	9 (4.0%)	8 (7.5%)	4 (2.9%)	3 (3.3%)	45 (6.1%)		
>5–16 years	6 (3.4%)	4 (1.8%)	0 (0.0%)	0 (0.0%)	2 (2.2%)	12 (1.6%)		
>16–59 years	3 (1.7%)	8 (3.5%)	3 (2.8%)	13 (9.6%)	1 (1.1%)	28 (3.8%)		
≥60 years	14 (7.9%)	13 (5.7%)	13 (12.1%)	7 (5.1%)	4 (4.3%)	51 (6.9%)		
Missing age	0 (0.0%)	1 (0.4%)	3 (2.8%)	2 (1.5%)	3 (3.3%)	9 (1.2%)		
Season, n (%)							<0.001	0.483 (0.454–0.515)
Winter	9 (5.1%)	100 (44.1%)	85 (79.4%)	119 (87.5%)	68 (73.9%)	381 (51.6%)		
Spring	40 (22.6%)	10 (4.4%)	8 (7.5%)	16 (11.8%)	13 (14.1%)	87 (11.8%)		
Summer	93 (52.5%)	6 (2.6%)	11 (10.3%)	0 (0.0%)	1 (1.1%)	111 (15.0%)		
Autumn	35 (19.8%)	111 (48.9%)	3 (2.8%)	1 (0.7%)	10 (10.9%)	160 (21.7%)		
Clinical service, n (%)							<0.001	0.254 (0.235–0.300)
Infectious diseases and allergy pediatric department	141 (79.7%)	110 (48.5%)	45 (42.1%)	43 (31.6%)	41 (44.6%)	380 (51.4%)		
Neonatology	1 (0.6%)	37 (16.3%)	21 (19.6%)	51 (37.5%)	15 (16.3%)	125 (16.9%)		
Pediatric medical ICU	4 (2.3%)	26 (11.5%)	9 (8.4%)	13 (9.6%)	21 (22.8%)	73 (9.9%)		
Other pediatric wards	14 (7.9%)	33 (14.5%)	14 (13.1%)	8 (5.9%)	8 (8.7%)	77 (10.4%)		
Adult medical ICU	14 (7.9%)	9 (4.0%)	8 (7.5%)	1 (0.7%)	0 (0.0%)	32 (4.3%)		
Non-acute medical service adult	3 (1.7%)	5 (2.2%)	5 (4.7%)	6 (4.4%)	7 (7.6%)	26 (3.5%)		
Emergency adult department	0 (0.0%)	7 (3.1%)	5 (4.7%)	14 (10.3%)	0 (0.0%)	26 (3.5%)		

### 3.3. Multivariable Binary Logistic Regression of Factors Associated with RSV Detection (Table 3)

To adjust for major potential confounders, a multivariable binary logistic regression model was fitted using RSV positivity (detected versus not detected) as the dependent variable. The model included age group, year of sampling, hospital service, assay type and season.

After adjustment, RSV detection remained strongly associated with sampling year, with significantly lower odds than in 2021 for all subsequent years: 2022 (aOR 0.32, 95% CI 0.24–0.42; *p* < 0.001), 2023 (aOR 0.12, 95% CI 0.09–0.17; *p* < 0.001), 2024 (aOR 0.22, 95% CI 0.15–0.33; *p* < 0.001), and 2025 (aOR 0.20, 95% CI 0.14–0.28; *p* < 0.001). Using infants aged 1–6 months as the reference group, the odds of RSV detection decreased significantly with increasing age, particularly among children aged 6 months to 2 years (aOR 0.54, 95% CI 0.41–0.71; *p* < 0.001), 2–5 years (aOR 0.33, 95% CI 0.22–0.48; *p* < 0.001), and 5–16 years (aOR 0.10, 95% CI 0.05–0.19; *p* < 0.001), whereas no significant difference was observed in neonates.

Regarding hospital setting, only pediatric intensive care was independently associated with lower odds of RSV detection compared with the Pediatrics I ward (aOR 0.54, 95% CI 0.39–0.73; *p* < 0.001), while no significant independent association was found for the other services. In addition, use of the GX assay remained associated with higher odds of RSV positivity compared with FR (aOR 1.96, 95% CI 1.33–2.89; *p* = 0.001). A clear seasonal pattern also persisted after adjustment: compared with winter, the odds of RSV detection were significantly lower in spring (aOR 0.22, 95% CI 0.16–0.29; *p* < 0.001), summer (aOR 0.27, 95% CI 0.20–0.36; *p* < 0.001), and autumn (aOR 0.55, 95% CI 0.43–0.71; *p* < 0.001). Overall, the model was statistically significant (*p* < 0.001) and showed moderate explanatory performance, with a pseudo-R^2^ of 0.263.

**Table 3:** Multivariable binary logistic regression of factors associated with RSV detection. Model adjusted for age, year, hospital service, assay type, and season. Included observations: n = 4409; RSV-positive cases: n = 730.

Variable	Category	Adjusted OR (aOR)	95% CI	*p*-Value
Age	1–6 months	Reference	—	—
	Neonates vs. 1–6 months	0.83	0.63–1.08	0.155
	6 months–2 years vs. 1–6 months	0.54	0.41–0.71	<0.001
	2–5 years vs. 1–6 months	0.33	0.22–0.48	<0.001
	5–16 years vs. 1–6 months	0.10	0.05–0.19	<0.001
	16–59 years vs. 1–6 months	0.08	0.00–2.67	0.156
	≥60 years vs. 1–6 months	0.14	0.00–4.91	0.279
Age	1–6 months16–59 years	Reference	—	—
	≥60 ans vs. 16–59 ans	1.83	1.14–2.95	0.013
**Year**	2021	Reference	—	—
	2022 vs. 2021	0.32	0.24–0.42	<0.001
	2023 vs. 2021	0.12	0.09–0.17	<0.001
	2024 vs. 2021	0.22	0.15–0.33	<0.001
	2025 vs. 2021	0.20	0.14–0.28	<0.001
**Hospital service**	Pediatrics I	Reference	—	—
	Neonatology vs. Pediatrics I	1.18	0.86–1.61	0.305
	Pediatric medical intensive care unit vs. Pediatrics I	0.54	0.39–0.73	<0.001
	Other pediatric wards vs. Pediatrics I	0.88	0.64–1.22	0.451
	Emergency department vs. Pediatrics I	0.71	0.02–25.32	0.852
	Adult medical intensive care unit vs. Pediatrics I	0.28	0.01–9.89	0.487
	Other adult wards vs. Pediatrics I	0.42	0.01–14.67	0.631
**Assay type**	FR	Reference	—	—
	GX vs. FR	1.96	1.33–2.89	0.001
**Season**	Winter	Reference	—	—
	Spring vs. Winter	0.22	0.16–0.29	<0.001
	Summer vs. Winter	0.27	0.20–0.36	<0.001
	Autumn vs. Winter	0.55	0.43–0.71	<0.001

### 3.4. RSV Co-Infection

Co-infection analysis was performed for samples tested using the Filmarray respiratory panel RP 2.1 plus (n = 665 RSV-positives). Among these, 53.3% (355/665) were mono-infections and 46.7% (310/665) were co-infections (n = 310).

Co-infections were significantly more frequent in children (48.5%, 287/592) than in adults (31.5%, 23/73) (*p* < 0.001) and decreased progressively with age (*p* < 0.001).

Human rhinovirus/enterovirus was the most frequently co-detected pathogen, found in 198 of 310 co-infections (63.9%), followed by adenovirus (45/310, 14.5%), parainfluenza viruses (35/310, 11.2%), SARS-CoV-2 (33/310, 10.6%), and seasonal coronaviruses (19/310, 6.1%).

### 3.5. Seasonal Trends of RSV Infection

Seasonal distribution varied significantly across years (*p* < 0.001), with a strong effect size (Cramér’s V = 0.483, 95% CI 0.454–0.515). Overall, sample volume peaked in winter (35.1%, 1614/4604), followed by spring (25.2%, 1160/4604), autumn (20.3%, 936/4604), and summer (19.4%, 894/4604). Notably, 2021 showed an atypical pattern, with peak sampling in summer (34.9%, 261/748) and low winter activity (17.4%, 130/748). In contrast, 2023–2024 were winter-dominated (45.7% (483/1057); 52.2% (408/781)), with reduced autumn in 2023 (3.4%, 36/1057), reflecting COVID-19 non-pharmaceutical intervention (NPI) effects on respiratory testing flows.

**Figure 2:**
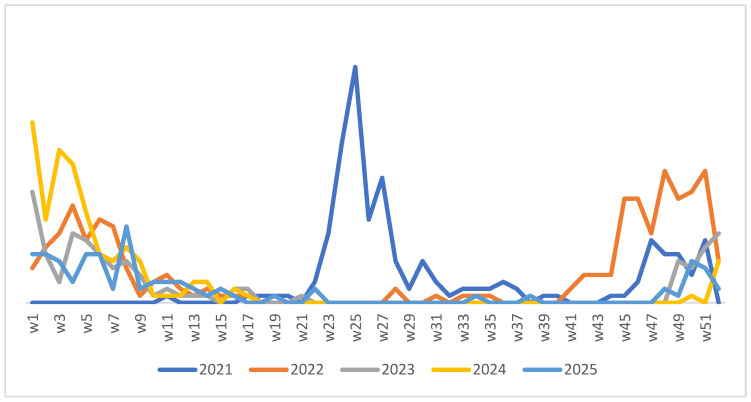
Weekly RSV positivity trends in SARI patients, CHU Ibn Sina-Rabat (2021–2025).

Weekly RSV trends (January 2021–December 2025; Figure 2) highlight interannual variability and re-establishment of typical winter dominance post-pandemic, with RSV positivity peaking at 51.6% in winter overall.

## 4. Discussion

RSV is a leading cause of SARIs worldwide, particularly in infants and older adults [2,3,4,6,26,34,36,37,38,39]. In our tertiary-care setting in Rabat (Ibn Sina University Hospital Center, 2021–2025), the positivity reached 16.1% (739/4604 SARI samples), with a predominantly pediatric burden (88.6% of RSV-positive cases) and a marked concentration among infants aged <6 months (70.4%). Annual detection rates fluctuated substantially (from 23.7% in 2021 to 10.1% in 2023; *p* < 0.001), with high co-infection frequencies (46.7%) and notable disruptions of the usual winter-dominant seasonality, including a pronounced summer peak in 2021 (52.5%) that progressively reverted to winter predominance by 2023–2025 (Figure 2). Together, these findings provide a multi-year, hospital-based baseline for RSV epidemiology in Rabat and highlight post-NPI shifts in circulation patterns in this understudied LMIC context.

Consistent with global evidence identifying RSV as the leading viral cause of bronchiolitis and severe lower respiratory tract infections in early life [2,5,8,29,40,41], most RSV detections in our cohort occurred in pediatric wards, particularly neonatology and general pediatrics, with infants in the first months of life being disproportionately affected, which likely reflects the combined effects of immature immune responses, narrow airway calibre, and limited pre-existing immunity in young infants. Although adults accounted for only 11.8% of RSV-positive cases, RSV remains clinically relevant in older age groups, with prior studies reporting RSV in approximately 4–10% of acute respiratory infections in individuals aged ≥60 years and substantial morbidity and mortality in high-risk populations [7,8,9,36,42]. Indeed, the relatively low number of RSV detections in adults may partly reflect diagnostic priorities during the COVID-19 pandemic, when SARS-CoV-2 singleplex PCR testing was frequently favoured over multiplex respiratory panels in adult care, potentially leading to under-ascertainment of RSV and viral co-infections in this age group.

We also confirmed through our results that year of sampling, age group, assay type, and season were independently associated with RSV detection, reinforcing the significant contribution of these factors to the observed epidemiological patterns beyond simple differences in patient distribution across hospital services. The markedly higher odds of RSV positivity in 2021, followed by lower odds from 2022 onwards, support the hypothesis of a substantial post-pandemic reshaping of RSV circulation, with persistence of interannual instability over the study period. The particularly high burden in infants aged 1–6 months underscores the well-recognized vulnerability of early infancy, whereas the progressive decline in odds with increasing age is consistent with the acquisition of partial immunity after repeated exposures. The persistence of a strong winter signal after adjustment confirms that, despite the transient disruption observed in 2021, RSV seasonality rapidly re-emerged as a dominant feature in our setting once pandemic-related restrictions were relaxed. The association between assay type and RSV positivity further suggests that diagnostic strategies including platform availability, sensitivity, and clinical indications for test use may have influenced case ascertainment and should be considered when interpreting temporal trends.

The particularly low detection rate observed in 2023 warrants cautious interpretation. During this period, our most frequently used platform, the FilmArray^^®^^ Respiratory Panel 2.1 Plus, was affected by temporary reagent shortages, which likely limited testing volume and may have contributed to an underestimation of RSV cases. In parallel, the relocation of several clinical departments in 2023, driven by the construction of a new hospital complex, may have reduced respiratory sampling and specimen referrals, thereby introducing additional logistical constraints and underscoring the importance of maintaining robust and resilient diagnostic capacity for accurate monitoring of RSV trends over time.

Nearly half of RSV-positive patients (46.5%) had at least one additional respiratory virus detected, most commonly human rhinovirus/enterovirus, adenovirus, SARS-CoV-2, and parainfluenza viruses. This is in line with previous pediatric studies reporting high rates of viral co-detections in hospitalised RSV cases [43,44,45,46,47]. Although the clinical impact of viral co-infections remains debated, several reports suggest that co-detections may modulate disease severity through enhanced airway inflammation or altered host immune responses. Our study was not designed to formally assess the prognostic impact of co-infections; nevertheless, the high co-detection rate observed supports the routine use of multiplex PCR assays for SARI in hospital settings to improve aetiological attribution, guide infection control measures, and refine clinical management. Beyond our cohort, published evidence from comparable pediatric settings has demonstrated that RSV infection in hospitalized infants is associated with longer hospital stays and a higher risk of respiratory failure compared to COVID-19, underscoring the greater clinical severity of RSV in this vulnerable age group [48].

In temperate regions, including North Africa, RSV typically exhibits a winter-dominant seasonal pattern. In our cohort, detection rates were highest in winter (48.3%) and autumn, but 2021 was characterised by an atypical summer peak, similar to out-of-season resurgences reported in multiple countries after the relaxation of COVID-19 non-pharmaceutical interventions [49,50,51,52,53,54]. These atypical epidemics have been attributed to a temporary “immunity gap” resulting from reduced viral exposure during 2020–2021, leading to an accumulation of susceptible young children [55,56,57]. In Rabat, RSV circulation progressively reverted to a winter-dominant pattern between 2022 and 2024, mirroring observations from other settings as population immunity re-equilibrated.

As part of our quality assurance programme, we performed a prospective, head-to-head comparison of the FilmArray^^®^^ Respiratory Panel 2.1 Plus and the Xpert^^®^^ Xpress SARS-CoV-2/Flu/RSV on SARI samples collected between 6 December 2023 and 28 February 2024. The results were fully concordant between platforms, with no discordant findings, confirming their analytical reliability and supporting their interchangeable use according to clinical needs and operational constraints. This high level of concordance reduces the likelihood that platform-related analytical differences explain the temporal trends observed over the study period.

Until 2023, Morocco lacked a dedicated national RSV surveillance system, and RSV monitoring was primarily integrated into sentinel networks focused on influenza and, more recently, COVID-19. In addition, Rabat’s Mediterranean–Atlantic climate, characterised by mild winters and relatively high humidity, may favour prolonged RSV circulation compared with colder continental climates, although dedicated studies are needed to clarify these associations. Finally, the preferential use of SARS-CoV-2 singleplex PCR in adult care during the early pandemic phase likely contributed to under-detection of RSV and viral co-infections in older patients and may have biased age-specific estimates.

Despite these constraints, our study provides a detailed, multi-year description of RSV epidemiology in a major tertiary care centre in Morocco across both the pandemic and post-pandemic periods. Given the recent introduction of maternal RSV vaccines and long-acting monoclonal antibodies for infants, a robust understanding of local RSV seasonality, age distribution, and co-infection patterns is crucial to inform optimal prevention strategies in low- and middle-income countries [5,6,7,34,44,45,46,47]. Our findings support the need to sustain multiplex PCR capacity, to broaden RSV surveillance beyond sentinel pediatric sites, and to strengthen preparedness for seasonal RSV epidemics in Morocco.

### 4.1. Limitations

Our investigation offers valuable perspectives on RSV epidemiology within our specific context. Yet it is important to recognize certain inherent constraints. Initially, the retrospective nature of the study may have introduced information bias, especially concerning incomplete demographic and clinical data. In addition, the heterogeneous testing strategy across years and hospital services may have introduced selection bias, as patients were not assessed using a fully uniform diagnostic approach throughout the study period. Furthermore, the lack of pre-pandemic RSV surveillance at our institution restricts the evaluation of long-term trends and the comprehensive effect of COVID-19 on RSV transmission [49,50,51,52,53,54,55,56]. Despite this, pre-pandemic Moroccan data indicated significant RSV detection in hospitalized SARI cases at CHU Ibn Sina (12%; 15/147, 2015–2016) [30] and within national sentinel ILI/SARI surveillance (17.9%; 359/2009, 2014–2016), thereby offering partial baseline context [39]. Finally, the temporary suspension of the FilmArray RP2.1plus system in 2023, coupled with the increased dependence on targeted GeneXpert SARS-CoV-2/Flu/RSV testing, may have led to an underestimation of RSV circulation and co-infections; analogous diagnostic deficiencies have been documented globally during the pandemic [52,55].

### 4.2. Perspectives

Strengthening RSV surveillance and prevention is now widely recognized as a major global public health priority [37,38]. In Morocco, enhancing molecular surveillance within existing respiratory virus monitoring systems would facilitate the early detection of changes in epidemic dynamics, including unusual off-season peaks [50,51,52,53,54,55]. The implementation of standardized RSV reporting, aligned with WHO and ECDC respiratory virus surveillance frameworks, would further strengthen preparedness and improve situational awareness [56,57]. Beyond surveillance, prevention strategies must also adapt. Maternal RSV vaccination and long-acting monoclonal antibodies for infants represent promising approaches to reduce morbidity in vulnerable populations [37,38]. Assessing their feasibility and optimal integration into the Moroccan setting will be essential, particularly given the substantial burden observed among infants younger than six months [2,5,40]. Future studies should further investigate RSV genotype circulation, climatic determinants of transmission [49], and the contribution of co-infections to disease severity [43,44,45,46,47], building on the growing evidence that RSV remains the leading viral cause of severe pneumonia in young children, as confirmed by recent comprehensive reviews [36]. In addition, expanding multiplex PCR testing to adult wards and intensive care units would provide a more comprehensive understanding of RSV epidemiology across age groups. As RSV prevention enters a new era, emerging real-world evidence suggests that the reduction in RSV-related emergency department visits and hospital admissions associated with long-acting monoclonal antibodies and maternal vaccination is largely dependent on the level of uptake and effective coverage achieved in target populations [58].

## 5. Conclusions

In this study, we characterized RSV epidemiology among 4604 pediatric and adult SARI patients at Ibn Sina University Hospital Center (Rabat, Morocco; 2021–2025). RSV positivity was 16.0% (739/4604), predominantly affecting infants <6 months (70.4% of cases) while remaining clinically relevant in adults. Co-infections occurred in 46.7% of FilmArray-tested positives, underscoring the multiplex PCR value. Seasonality shifted from a 2021 summer peak (52.5%) to typical winter dominance post-2023, reflecting COVID-19 NPI disruption and immunity debt. These findings establish the first comprehensive Moroccan baseline for RSV surveillance, informing optimal timing and targeting of maternal vaccines and long-acting monoclonal antibodies in this understudied LMIC (low- and middle-income country) setting.

## Figures and Tables

**Table 1:** Baseline characteristics of patients hospitalized for SARI at the University Hospital Center, Rabat, Morocco, 2021–2025; N = 4604: (a) Annual distribution of SARI hospitalizations by year (2021, n = 748; 2022, n = 1383; 2023, n = 1057; 2024, n = 781; 2025, n = 635). (b) *Gender, age category, age group*, *seasonal distribution* and *hospital service * of SARI hospitalizations over the study period; percentages are calculated within each year using the corresponding annual denominator (2021, n = 748; 2022, n = 1383; 2023, n = 1057; 2024, n = 781; 2025, n = 635 *and* Total (n = 4604)). *p*-values were derived from Pearson’s chi-square test for categorical variables and the Kruskal–Wallis test for continuous age. Effect sizes are reported as Cramér’s V.

Characteristics	2021 (n = 748)	2022 (n = 1383)	2023 (n = 1057)	2024 (n = 781)	2025 (n = 635)	Total (n = 4604)	*p*-Value	Effect Size (Cramér’s V)
Gender, n (%)							0.363	0.031
Female	326 (43.6%)	596 (43.1%)	481 (45.5%)	366 (46.9%)	295 (46.5%)	2064 (44.8%)		
Male	422 (56.4%)	787 (56.9%)	576 (54.5%)	415 (53.1%)	340 (53.5%)	2540 (55.2%)		
Age category, n (%)							<0.001	0.135
Children	347 (46.4%)	627 (45.3%)	582 (55.1%)	353 (45.2%)	407 (64.1%)	2316 (50.3%)		
Adults	401 (53.6%)	756 (54.7%)	475 (44.9%)	428 (54.8%)	228 (35.9%)	2288 (49.7%)		
Age group, n (%)							<0.001	0.136
0–31 days	38 (5.1%)	125 (9.0%)	126 (11.9%)	105 (13.4%)	117 (18.4%)	511 (11.1%)		
1–6 months	90 (12.0%)	233 (16.8%)	240 (22.7%)	134 (17.2%)	90 (14.2%)	787 (17.1%)		
6 months–2 years	117 (15.6%)	147 (10.6%)	105 (9.9%)	55 (7.0%)	108 (17.0%)	532 (11.6%)		
>2–5 years	63 (8.4%)	61 (4.4%)	58 (5.5%)	21 (2.7%)	35 (5.5%)	238 (5.2%)		
>5–16 years	35 (4.7%)	54 (3.9%)	44 (4.2%)	35 (4.5%)	49 (7.7%)	217 (4.7%)		
>16–59 years	203 (27.1%)	373 (27.0%)	242 (22.9%)	179 (22.9%)	86 (13.5%)	1083 (23.5%)		
≥60 years	187 (25.0%)	369 (26.7%)	222 (21.0%)	220 (28.2%)	43 (6.8%)	1041 (22.6%)		
Missing age	15 (2.0%)	21 (1.5%)	20 (1.9%)	32 (4.1%)	107 (16.9%)	195 (4.2%)		
Season, n (%)							<0.001	0.250
Winter	130 (17.4%)	380 (27.5%)	483 (45.7%)	408 (52.2%)	213 (33.5%)	1614 (35.1%)		
Spring	184 (24.6%)	211 (15.3%)	392 (37.1%)	245 (31.4%)	128 (20.2%)	1160 (25.2%)		
Summer	261 (34.9%)	312 (22.6%)	146 (13.8%)	67 (8.6%)	108 (17.0%)	894 (19.4%)		
Autumn	173 (23.1%)	480 (34.7%)	36 (3.4%)	61 (7.8%)	186 (29.3%)	936 (20.3%)		
Clinical service, n (%)							<0.001	0.229
Infectious diseases and allergy pediatric department	279 (37.3%)	324 (23.4%)	291 (27.5%)	119 (15.2%)	220 (34.6%)	1233 (26.8%)		
Neonatology	2 (0.3%)	84 (6.1%)	77 (7.3%)	94 (12.0%)	50 (7.9%)	307 (6.7%)		
Pediatric medical ICU	39 (5.2%)	126 (9.1%)	93 (8.8%)	84 (10.8%)	102 (16.1%)	444 (9.6%)		
Non-acute medical service pediatric	27 (3.6%)	93 (6.7%)	121 (11.4%)	56 (7.2%)	35 (5.5%)	332 (7.2%)		
Adult medical ICU	317 (42.4%)	422 (30.5%)	163 (15.4%)	95 (12.2%)	25 (3.9%)	1022 (22.2%)		
Non-acute medical service adult	63 (8.4%)	189 (13.7%)	142 (13.4%)	150 (19.2%)	201 (31.7%)	745 (16.2%)		
Emergency adult department	21 (2.8%)	145 (10.5%)	170 (16.1%)	183 (23.4%)	2 (0.3%)	521 (11.3%)		

## Data Availability

The data supporting this study include patient demographic and laboratory information and cannot be made publicly available due to privacy and ethical restrictions, in accordance with institutional and national regulations. However, de-identified data may be made available to journal reviewers for evaluation purposes during the peer-review process upon reasonable request to the corresponding author, subject to institutional approval.

## Abbreviations

The following abbreviations are used in this manuscript:

ADV	Adenovirus
ARI	Acute Respiratory Infection
UHC	University Hospital Center
CI	Confidence Interval
CoV	Coronavirus
COVID-19	Coronavirus disease 2019–related coronavirus
CoV 229E	Human Coronavirus 229E
CoV HKU1	Human Coronavirus HKU1
CoV NL63	Human Coronavirus NL63
CoV OC43	Human Coronavirus OC43
Ct	Cycle Threshold
CVL	Central Virology Laboratory
EV	Enterovirus
Flu A	Influenza A
Flu B	Influenza B
hMPV	Human Metapneumovirus
HRV	Human Rhinovirus
HRV/EV	Human Rhinovirus/Enterovirus
IAV	Influenza A Virus
IBV	Influenza B Virus
ICU	Intensive Care Unit
ILI	Influenza-Like Illness
LMIC	Low- and Middle-Income Country
MERS-CoV	Middle East Respiratory Syndrome Coronavirus
NPIs	Non-Pharmaceutical Interventions
PCR	Polymerase Chain Reaction
PHEIC	Public Health Emergency of International Concern
RSV	Respiratory Syncytial Virus
RT-PCR	Reverse Transcription Polymerase Chain Reaction
SARI	Severe Acute Respiratory Infection
SARS-CoV-2	Severe Acute Respiratory Syndrome Coronavirus 2
UTM	Universal Transport Medium
WHO	World Health Organization

## References

[1] Hodgson D., Pebody R., Panovska-Griffiths J., Baguelin M., Atkins K.E. (2020). Evaluating the next generation of RSV intervention strategies: A mathematical modelling study and cost-effectiveness analysis. BMC Med..

[2] Li Y., Wang X., Blau D.M., Caballero M.T., Feikin D.R., Gill C.J., Madhi S.A., Omer S.B., Simões E.A.F., Campbell H. (2022). Global, regional, and national disease burden estimates of acute lower respiratory infections due to respiratory syncytial virus in children younger than 5 years in 2019: A systematic analysis. Lancet.

[3] Martinón-Torres F., Navarro-Alonso J.A., Garcés-Sánchez M., Soriano-Arandes A. (2023). The path towards effective respiratory syncytial virus immunization policies: Recommended actions. Arch. Bronconeumol..

[4] Munro A.P.S., Martinón-Torres F., Drysdale S.B., Faust S.N. (2023). The disease burden of respiratory syncytial virus in infants. Curr. Opin. Infect. Dis..

[5] Mazela J., Jackowska T., Czech M., Helwich E., Martyn O., Aleksiejuk P., Smaga A., Tkacz A., Glazewska J., Wysocki J. (2025). Clinical burden and healthcare utilization associated with hospitalizations of RSV-infected Polish children during the 2022/23 season. Viruses.

[6] Abrams E.M., Doyon-Plourde P., Davis P., Davis P., Brousseau N., Irwin A., Siu W., Killikelly A. (2024). Burden of disease of RSV in infants, children and pregnant women and people. Can. Commun. Dis. Rep..

[7] Langley J.M., Bianco V., Domachowske J.B., Madhi S.A., Stoszek S.K., Zaman K., Bueso A., Ceballos A., Cousin L., D’Andrea U. (2022). Incidence of respiratory syncytial virus lower respiratory tract infections during the first 2 years of life: A prospective study across diverse global settings. J. Infect. Dis..

[8] Rameix-Welti M.A. (2024). Towards widespread prevention of respiratory syncytial virus (RSV) infections in children and the elderly. J. Pédiatrie Puéricult..

[9] See K.C. (2023). Vaccination for respiratory syncytial virus: A narrative review and primer for clinicians. Vaccines.

[10] Yu J., Liu N., Zhu Y., Wang W., Fan X., Yuan X., Xu J., Zheng B., Luan L. (2024). Comparative study on the epidemiological characteristics and hazards of respiratory syncytial virus and influenza virus infections among elderly people. BMC Infect. Dis..

[11] Roumanes D., Falsey A.R., Quataert S., Secor-Socha S., Lee F.E., Yang H., Bandyopadhyay S., Holden-Wiltse J., Topham D.J., Walsh E.E. (2018). T-cell responses in adults during natural respiratory syncytial virus infection. J. Infect. Dis..

[12] Alfano F., Bigoni T., Caggiano F.P., Papi A. (2024). Respiratory syncytial virus infection in older adults: An update. Drugs Aging.

[13] Rzymski P., Poniedziałek B., Zarębska-Michaluk D., Tomasiewicz K., Flisiak R. (2025). High seroprevalence and high risk: Why are older adults more prone to respiratory syncytial virus?. J. Virol..

[14] Englund J., Feuchtinger T., Ljungman P. (2011). Viral infections in immunocompromised patients. Biol. Blood Marrow Transplant..

[15] Lanari M., Vandini S., Capretti M.G., Lazzarotto T., Faldella G. (2014). Respiratory syncytial virus infections in infants affected by primary immunodeficiency. J. Immunol. Res..

[16] Nduaguba S.O., Tran P.T., Choi Y., Winterstein A.G. (2023). Respiratory syncytial virus reinfections among infants and young children in the United States, 2011–2019. PLoS ONE.

[17] Trento A., Galiano M., Videla C., Carballal G., García-Barreno B., Melero J.A., Palomo C. (2015). Major changes in the G protein of human respiratory syncytial virus isolates introduced by a duplication of 60 nucleotides. Sci. Rep..

[18] Cantú-Flores K., Rivera-Alfaro G., Muñoz-Escalante J.C., Noyola D.E. (2022). Global distribution of respiratory syncytial virus A and B infections. Pathog. Glob. Health.

[19] Duvvuri V.R., Granados A., Rosenfeld P., Bahl J., Eshaghi A., Gubbay J.B. (2015). Genetic diversity and evolutionary insights of respiratory syncytial virus A ON1 genotype: Global and local transmission dynamics. J. Virol..

[20] Yoshihara K., Le M.N., Okamoto M., Wadagni A.C.A., Nguyen H.A., Toizumi M., Pham E., Suzuki M., Nguyen A.T.T., Oshitani H. (2016). Association of RSV-A ON1 genotype with Increased Pediatric Acute Lower Respiratory Tract Infection in Vietnam. Sci Rep.

[21] Eshaghi A., Duvvuri V.R., Lai R., Nadarajah J.T., Li A., Patel S.N., Low D.E., Gubbay J.B. (2012). Genetic Variability of Human Respiratory Syncytial Virus A Strains Circulating in Ontario: A Novel Genotype with a 72 Nucleotide G Gene Duplication. PloS ONE.

[22] Branche A.R., Saiman L., Walsh E.E., Falsey A.R., Sieling W.D., Greendyke W., Peterson D.R., Vargas C.Y., Phillips M., Finelli L. (2022). Incidence of Respiratory Syncytial Virus Infection Among Hospitalized Adults, 2017-2020. Clinical infectious diseases: An official publication of the Infectious Diseases Society of America.

[23] Fodha I., Vabret A., Ghedira L., Seboui H., Chouchane S., Dewar J., Gueddiche N., Trabelsi A., Boujaafar N., Freymuth F. (2007). Respiratory syncytial virus infections in hospitalized infants: Association between viral load, virus subgroup, and disease severity. J. Med. Virol..

[24] Tramuto F., Maida C.M., Mazzucco W., Costantino C., Amodio E., Sferlazza G., Previti A., Immordino P., Vitale F. (2023). Molecular Epidemiology and Genetic Diversity of Human Respiratory Syncytial Virus in Sicily during Pre- and Post-COVID-19 Surveillance Seasons. Pathogens.

[25] Goya S., Lucion M.F., Shilts M.H., del Valle Juárez M., Gentile A., Mistchenko A.S., Viegas M., Das S.R. (2023). Evolutionary dynamics of respiratory syncytial virus in Buenos Aires: Viral diversity, migration, and subgroup replacement. Virus Evolution.

[26] Munoz N.I., Terstappen J., Baral R., Bardají A., Beutels P., Buchholz U.J., Cohen C., Crowe J.E., Cutland C.L., Eckert L. (2023). Respiratory syncytial virus prevention within reach: The vaccine and monoclonal antibody landscape. The Lancet. Infectious diseases.

[27] Ahmed K., Amine B.M., Ahmed O. (2025). Circulating viral respiratory pathogens as causative agents for severe acute respiratory infections in Morocco: A systematic review. Afr. J. Infect. Dis..

[28] Jroundi I., Mahraoui C., Benmessaoud R., Moraleda C., Tligui H., Seffar M., Kettani S.C., Benjelloun B.S., Chaacho S., Maaroufi A. (2014). The epidemiology and aetiology of infections in children admitted with clinical severe pneumonia to a university hospital in Rabat, Morocco. J. Trop. Pediatr..

[29] Lamrani Hanchi A., Guennouni M., Rachidi M., Benhoumich T., Bennani H., Bourrous M., Maoulainine F.M.R., Younous S., Bouskraoui M., Soraa N. (2021). Epidemiology of respiratory pathogens in children with severe acute respiratory infection and impact of the multiplex PCR FilmArray respiratory panel: A 2-year study. Int. J. Microbiol..

[30] Marcil S., Kabbaj H., Jroundi I., Barakat A., Mahraoui C., Kettani S., Zeggwagh A., Khouchoua M., Belefquih B., Seffar M. (2018). Epidemiology and diagnosis of severe acute viral respiratory infections in patients admitted at Ibn Sina University Hospital Rabat-Morocco. Dis. Disord..

[31] Ma Y., Fan S., Xi J. (2025). Recent updates regarding the management and treatment of pneumonia in pediatric patients: A comprehensive review. Infection.

[32] Edderdouri K., Kabbaj H., Laamara L., Lahmouddi N., Lamdarsi O., Zouaki A., El Amin G., Zirar J., Seffar M. (2023). Contribution of the FilmArray BioFire technology in the diagnosis of viral respiratory infections during the COVID-19 pandemic at Ibn Sina University Hospital Center in Rabat: Epidemiological study about 503 cases. Adv. Virol..

[33] bioMérieux Website [Internet] BIOFIRE^^®^^ Respiratory 2.1 et 2.1plus Panels. https://www.biomerieux.com/fr/fr/notre-offre/produits-clinique/biofire-respiratory-2-1-panels.html.

[34] Bally-von Passavant E.D., Joseph N., Kräutler N.J., McCarthy-Pontier D., Lüthi-Corridori G., Jaun F., Leuppi J.D., Boesing M. (2025). Burden and characteristics of RSV-associated hospitalizations in Switzerland: A nation-wide analysis from 2017 to 2023. Viruses.

[35] Xpert^^®^^ Xpress CoV-2/Flu/RSV Plus [Internet]. https://www.cepheid.com/fr-FR/tests/respiratory/xpert-xpress-cov-2-flu-rsv-plus.html.

[36] Abrams E.M., Doyon-Plourde P., Davis P., Lee L., Rahal A., Brousseau N., Siu W., Killikelly A. (2025). Burden of disease of respiratory syncytial virus in older adults and adults considered at high risk of severe infection. Can. Commun. Dis. Rep..

[37] Sanz-Muñoz I., Sánchez-De Prada L., Castrodeza-Sanz J., Eiros J.M. (2024). Microbiological and epidemiological features of respiratory syncytial virus. Rev. Esp. Quimioter..

[38] Yassine H.M., Sohail M.U., Younes N., Nasrallah G.K. (2020). Systematic review of the respiratory syncytial virus prevalence, genotype distribution, and seasonality in children from the Middle East and North Africa region. Microorganisms.

[39] Bimouhen A., El Falaki F., Ihazmad H., Regragui Z., Benkerroum S., Barakat A. (2016). Circulation of Respiratory Syncytial Virus in Morocco during 2014–2016: Findings from a sentinel-based virological surveillance system for 413 influenza. East. Mediterr. Health J..

[40] Lamrani Hanchi A., Guennouni M., Ben Houmich T., Echchakery M., Draiss G., Rada N., Younous S., Bouskraoui M., Bouskraoui N. (2022). Changes in the epidemiology of respiratory pathogens in children during the COVID-19 pandemic. Pathogens.

[41] Shi T., McAllister D.A., O’Brien K.L., Simoes E.A.F., Madhi S.A., Gessner B.D., Polack F.P., Balsells E., Acacio S., Aguayo C. (2017). Global, regional, and national disease burden estimates of acute lower respiratory infections due to respiratory syncytial virus in young children in 2015: A systematic review and modelling study. Lancet.

[42] Loubet P., Lenzi N., Valette M., Foulongne V., Krivine A., Houhou N., Lagathu G., Rogez S., Alain S., Duval X. (2017). Clinical characteristics and outcome of respiratory syncytial virus infection among adults hospitalized with influenza-like illness in France. Clin. Microbiol. Infect..

[43] Huguenin A., Moutte L., Renois F., Leveque N., Talmud D., Abely M., Nguyen Y., Carrat F., Andreoletti L. (2012). Broad respiratory virus detection in infants hospitalized for bronchiolitis by use of a multiplex RT-PCR DNA microarray system. J. Med. Virol..

[44] Li Y., Pillai P., Miyake F., Nair H. (2020). The role of viral co-infections in the severity of acute respiratory infections among children infected with respiratory syncytial virus (RSV): A systematic review and meta-analysis. J. Glob. Health.

[45] Martin E.T., Kuypers J., Wald A., Englund J.A. (2012). Multiple versus single virus respiratory infections: Viral load and clinical disease severity in hospitalized children. Influenza Other Respir. Viruses.

[46] Pacheco G.A., Gálvez N.M.S., Soto J.A., Andrade C.A., Kalergis A.M. (2021). Bacterial and viral coinfections with the human respiratory syncytial virus. Microorganisms.

[47] Miron V.D., Raianu R.O., Filimon C., Craiu M. (2024). Clinical differences between SARS-CoV-2 and RSV infections in infants: Findings from a case-control study. Viruses.

[48] Alkharsah K.R. (2022). The scope of respiratory syncytial virus infection in a tertiary hospital in the Eastern Province of Saudi Arabia and the change in seasonal pattern during and after the COVID-19 pandemic. Medicina.

[49] Marriott D., Beresford R., Mirdad F., Stark D., Glanville A., Chapman S., Harkness J., Dore G.J., Andresen D., Matthews G.V. (2021). Marked and concomitant decline in severe acute respiratory syndrome coronavirus 2 (SARS-CoV-2) and other respiratory viruses among symptomatic patients following introduction of public health interventions in Australia: Data from St Vincent’s Hospital and associated screening clinics, Sydney, New South Wales. Clin. Infect. Dis..

[50] Delestrain C., Danis K., Hau I., Behillil S., Billard M.N., Krajten L., Cohen R., Bont L., Epaud R. (2021). Impact of COVID-19 social distancing on viral infection in France: A delayed outbreak of RSV. Pediatr. Pulmonol..

[51] Huang Q.S., Wood T., Jelley L., Jennings T., Jefferies S., Daniells K., Nesdale A., Dowell T., Turner N., Campbell-Stokes P. (2021). Impact of the COVID-19 nonpharmaceutical interventions on influenza and other respiratory viral infections in New Zealand. Nat Commun.

[52] Lumley S.F., Richens N., Lees E., Cregan J., Kalimeris E., Oakley S., Morgan M., Segal S., Dawson M., Walker A.S. (2022). Changes in pediatric respiratory infections at a UK teaching hospital 2016–2021: Impact of the SARS-CoV-2 pandemic. J Infect.

[53] Treggiari D., Pomari C., Zavarise G., Piubelli C., Formenti F., Perandin F. (2024). Characteristics of respiratory syncytial virus infections in children in the post-COVID seasons: A Northern Italy hospital experience. Viruses.

[54] Ando H., Ahmed W., Iwamoto R., Ando Y., Okabe S., Kitajima M. (2023). Impact of the COVID-19 pandemic on the prevalence of influenza A and respiratory syncytial viruses elucidated by wastewater-based epidemiology. Sci. Total Environ..

[55] European Centre for Disease Prevention and Control (2025). European Respiratory Virus Surveillance Summary (ERVISS) [Internet]. https://www.ecdc.europa.eu/en/publications-data/european-respiratory-virus-surveillance-summary-erviss.

[56] Trigueros Montes J.B., Montes D., Miele A., Baik-Han W., Gulati G., Lew L.Q. (2024). The impact of COVID-19 pandemic on respiratory syncytial virus infection in children. Pulm. Med..

[57] van Summeren J., Meijer A., Aspelund G., Casalegno J.S., Erna G., Hoang U., Lina B., de Lusignan S., Teirlinck A.C., Thors V. (2021). Low levels of respiratory syncytial virus activity in Europe during the 2020/21 season: What can we expect in the coming summer and autumn/winter?. Eurosurveillance.

[58] Perramon-Malavez A., Chiaretti A., Coma E., Craiu M., Foster S., Leonard P., Marlow R., Thors V., Martínez-Marcos M., Mendioroz J. (2026). Real-world impact of nirsevimab immunisation and maternal RSV vaccination against respiratory disease on emergency department attendances and admissions: A multinational retrospective analysis. Lancet Reg. Health Eur..

